# Modification and verification of the Infant–Toddler Meaningful Auditory Integration Scale: a psychometric analysis combining item response theory with classical test theory

**DOI:** 10.1186/s12955-020-01620-9

**Published:** 2020-11-13

**Authors:** Fengling Yang, Fei Zhao, Yun Zheng, Gang Li

**Affiliations:** 1 Hearing Center/Hearing and Speech Laboratory, Department of Otorhinolaryngology Head and Neck Surgery, West China Hospital, Sichuan University, Sixth Floor of Clinical Medicine Building, No 16, 3rd part, Renmin Road South, Wuhou District, Chengdu, China; 2 Center for Speech and Language Therapy and Hearing Science, Cardiff School of Sport and Health Sciences, Cardiff Metropolitan University, Cardiff, UK

**Keywords:** Infant–Toddler Meaningful Integration Scale, Early prelingual auditory development, Item response theory, Classical test theory

## Abstract

**Background:**

Early prelingual auditory development (EPLAD) is a fundamental and important process in the speech and language development of infants and toddlers. The Infant–Toddler Meaningful Auditory Integration Scale (ITMAIS) is a widely used measurement tool for EPLAD, however it has not yet undergone a comprehensive psychometric analysis. The aim of this research was to modify and verify the psychometric properties of ITMAIS using a combination of Item Response Theory (IRT) and Classical Test Theory (CTT).

**Methods:**

Stage 1—1730 children were retrospectively recruited to enable the application of an IRT model, specifically the graded response model, to modify the ITMAIS. Stage 2—another 450 infants and toddlers with normal hearing or permanent hearing loss before auditory intervention were recruited to verify the psychometric properties of the modified ITMAIS (ITMAIS-m) using the CTT method.

**Results:**

Using the metric of the graded response model, by removing item 2 from the ITMAIS, ITMAIS-m demonstrated discrimination parameters ranging from 3.947 to 5.431, difficulty parameters from − 1.146 to 1.150, item information distributed between 4.798 and 9.259 and a test information score of 48.061. None of the items showed differential item functioning. ITMAIS-m was further verified in Stage 2, showing Cronbach’s α of 0.919 and item-total correlations ranging from 0.693 to 0.851. There was good convergent validity of ITMAIS-m with other auditory outcome measure (r = 0.932) and pure tone average thresholds (r ranging from − 0.670 to − 0.909), as well as a high ability to discriminate between different hearing grades (Cohen d ranging from 0.41 to 5.83).

**Conclusions:**

The ITMAIS-m is a reliable and valid tool for evaluating EPLAD in infants and toddlers, which can be efficiently and precisely applied in clinical practice. The combined use of IRT and CTT provides a powerful means to modify psychometrically robust scales aimed at childhood auditory outcome measurements.

## Introduction

According to a recent WHO report (2019), 34 million children younger than 14 years of age have a disabling hearing loss [1]. Childhood hearing loss is a public health concern, with its deleterious influence on an individual’s speech and language development, educational performance and social-emotional development, as well as the heavy financial burden to health care systems and society [2–4]. In an attempt to maximize speech and language competence in hearing-impaired children, the Joint Committee on Infant Hearing (JCIH) issued guidelines for Early Hearing Detection and Intervention (EHDI) programs for infants in 2000, and later were updated in 2007 and 2019. This emphasizes the importance of early auditory evaluation and intervention [5–7].

Apart from various tests assessing hearing thresholds, auditory outcome measures also play an important role in the auditory evaluation of children [8]. Auditory outcome measures collect information in regards to a child’s ability to detect, discriminate, identify and comprehend sounds, information that is almost impossible to obtain from audiometric tests [9]. There are a number of auditory outcome measurement tools available. The Infant–Toddler Meaningful Integration Scale (ITMAIS) is one that is able to evaluate infants and toddlers’ early prelingual auditory development (EPLAD) in aspects of detection, discrimination and identification of sounds. This is achieved from parental observation reports on children’s auditory behaviors in daily routines [9–12]. With the advantage of time-saving and freedom from reliance on test conditions and compliance of children, the ITMAIS has been translated into many different languages and widely used for EPLAD evaluation [13–16]. Moreover, its usefulness is reinforced by its high Cronbach’s alpha, split-half reliability and item-total correlation scores in the different language versions, which highlight the psychometric properties of the tool [13–17].

It is noteworthy that the satisfactory psychometric outcomes with ITMAIS have been assessed using classical test theory (CTT) [18]. CTT hypothesizes that observed score is the linear combination of underlying true score and random error [19]. The true score, which is essentially the expected value (e.g. the EPLAD) intended to measure by infinite administrations of the same assessment (e.g. ITMAIS), could only be obtained when there is no random error in assessment [20]. Random error, the difference between the true score and observed score, is assumed to be normally distributed and uncorrelated with the true score. CTT mainly measures two kinds of psychometric parameters: reliability and validity [21]. Reliability concentrates on the consistency between the true score and observed score. The higher of the reliability, the higher ability of the observed score representing true score. Validity represents the capacity of a scale to assess what the scale intended to assess [19, 22]. With the advantage of easy-to-analyze, and the effectiveness in evaluating test–retest reliability and external structure of scale, CTT has been widely used to evaluate the psychometric characteristics of scales for decades of years.

In contrast to CTT, Item Response Theory (IRT) uses non-linear mathematical models, and estimates both item parameters and individual latent traits of subjects in a common scale [19, 23]. Different models used in IRT analyses vary in functional forms and the amount of item parameters estimated. Specifically, the item parameters estimated in the framework of IRT rely on the mathematical models instead of response proportions or item-total correlations. Furthermore, the estimated parameters are stable and independent from particular samples, provided the samples are drawn from the same population. However, before IRT modeling and parameter estimation, the fundamental assumptions (i.e., unidimensionality, local independence, monotonicity), as well as model fitting, should be evaluated in advance. Despite rigid assumptions before modeling and challenging mathematical requirements, IRT is gradually being applied to patient-reported outcome measures [18, 19, 24]. In light of the advantages and disadvantages of the two theories, an approach using a combination of both CTT and IRT has been suggested and implemented in current modification and validation of outcome measurements, as well as in the field of auditory-specific patient-reported outcome measures [20, 22, 25, 26].

Therefore, the present study aimed to combine IRT and CTT to form a comprehensive and complementary approach to the psychometric analysis of ITMAIS. The characteristics of each item of ITMAIS in a common scale were analyzed using the IRT, followed by modification by trimming away poorly performing items without affecting scale parameters. The psychometric properties of the modified ITMAIS (ITMAIS-m) were re-evaluated using the CTT framework.

## Materials and methods

### Study design

The present study comprised two stages. In Stage 1, a retrospective study was conducted to analyze and modify the ITMAIS using the IRT framework. In Stage 2, psychometric properties of ITMAIS-m were examined using a separate sample, and verified in the aspects of reliability and validity using CTT. In the process of validity evaluation, the relationships between the ITMAIS-m and individual pure tone average threshold (PTA) and hearing grades were examined. The study was conducted in accordance with the principles of the Declaration of Helsinki, and the study protocol was approved by the Biomedical Ethics Committee of West China Hospital of Sichuan University.

### Participants

In Stage 1, a total of 1983 Chinese children with different hearing grades and different types of hearing loss were recruited in the Hearing Center database of the West China Hospital of Sichuan University, Sichuan, China from Nov. 2006 to Jun. 2017. A total of 3404 ITMAIS assessments were undertaken before or after auditory intervention**.** Following exclusion of cases missing clinical data or item information, 1730 children (median age and interquartile range (IQR) 29.0 (17.6, 41.9) months) completed 3092 ITMAIS assessments (a total of 642 children assessed more than once) were included in the final statistical analysis.

In Stage 2, Chinese children with normal hearing or permanent hearing loss were recruited at the Hearing Center database from Jul. 2018 to Jun. 2019. Individuals with the possibility of a fluctuating hearing loss, confirmed auditory neuropathy spectrum disorder or other system disorder were excluded, eliminating any heterogeneous effects on ITMAIS-m assessment, and therefore on validity analysis. Participants in Stage 1 were not allowed to recruit in Stage 2. A total of 450 children (median age and IQR 5.7 (3.6, 9.3) months) provided 450 copies of the ITMAIS-m assessment (0 to 1 unanswered item was allowed) for analysis. Of the participants, 93 children were simultaneously assessed with a LittlEARS Auditory Questionnaire (LEAQ). Children in Stage 2 were subdivided into five age groups: 0–3.0 months, 3.1–6.0 months, 6.1–9.0 months, 9.1–16.0 months and 16.1–24.0 months.

### Assessment tools

The ITMAIS assessment tool used was based on the Chinese version translated by Zheng et al. [13] (as shown in Additional file 1). The first item relating to reliance on auditory instruments, was not suitable for assessing children without auditory intervention. As a consequence, assessment in the present study involved 9 items, with item 1 excluded. Through a structured interview with parents or caregivers that took typically 10 min, a trained audiologist scored the frequencies of meaningful auditory incidents in children observed in daily routines. Each item was scored 0 to 4, in which 0 represented incidents never observed, and 1, 2, 3, 4 respectively represented incidents rarely, occasionally, frequently and always observed. The total score was expressed as a percentage by dividing the actual score by the maximum score. ITMAIS-m was assessed in the same manner.

LEAQ is another structured interview questionnaire, assessing early auditory development in children under the age of 2 years [27]. Parents or caregivers in the present study were supported by an audiologist in completing the LEAQ to avoid any misunderstanding of questions. The total score was calculated by summing the number of items answering ‘yes’.

### Audiological tests

Children were subject to the auditory test battery following the ITMAIS-m or LEAQ assessment. Hearing grades and types were diagnosed by air and bone conduction of tone burst auditory brainstem responses, combined with otoacoustic emissions, acoustic immittance and behavioral audiometry. PTA was calculated using thresholds at 500, 1000, 2000 and 4000 Hz. Hearing grades were classified as mild, moderate, severe and profound hearing loss referring to PTA, according to the WHO criteria [28].

### Statistical analysis

#### Stage 1: Item analysis and modification of ITMAIS

Item analysis and modification of the ITMAIS in Stage 1, realized with the *Lavaan*,* Mokken*,* Mirt* and *Lordif* package in *R 3.5.3*, was guided by the psychometric evaluation plan recommended by Reeve et al. [29, 30].

#### Item responses and traditional statistic description

Frequencies of missing data, mean score and answer options of each item were calculated. Individuals with any unanswered item were analyzed and excluded in the analysis in Stage 1. Inter-item correlations between 0.2 and 0.8 were considered acceptable [31].

#### Assumptions checking before IRT modeling

The assumption of unidimensionality tests whether ITMAIS measures a single dominant latent trait—EPLAD. In the present study, the assumption was evaluated by combining exploratory factor analysis (EFA) and confirmatory factor analysis (CFA). The sample of Stage 1 was randomly split into two parts (i.e., Sample part 1: 1546 vs. Sample part 2: 1546 ITMAIS assessments), which were used to conduct EFA and CFA separately. In the approach of EFA, judged by eigenvalues (a ratio between factors > 4), explainable proportions of variance (> 25%) and factor loadings, main factors were extracted by principal factor solution under parallel analysis [29]. The results of CFA referred to indices with a series of criteria representing good fit: comparative fit index (CFI) > 0.95, Tucker–Lewis index (TLI) > 0.95, root mean square error of approximation (RMSEA) < 0.06 and standardized root mean residuals (SRMR) < 0.08 [29, 32].

In the present study, the local independence means that there should not be any relationship among item responses after conditioning on the level of EPLAD. This assumption was assessed with residual correlations obtained from the 1-factor CFA analysis. The correlation less than 0.1 was considered as eligible local independence [33, 34].

The monotonicity assumption signifies that the probability of endorsing a category of an item in ITMAIS increases when the level of EPLAD ascends. It was analyzed by judging from graphs plotting item step response function and item response function in the Mokken package [29].

#### IRT model fit and parameters evaluation

Among the various models in the IRT family, we chose the graded response model (GRM), with its flexibility for items with polytomous and ordered responses [29, 35, 36]. After confirming with three assumptions, item fit between the observed and expected responses under GRM was investigated. The *p* value of goodness-of-fit index *s-x*^2^ < 0.001 was considered with item misfit [29, 37].

Briefly, in the approach of GRM, the probability of a person *j* endorsing the category *k* or higher of an item *i* in ITMAIS is calculated as follows:

$$P \, (X_{i} \ge \, k|\theta_{j} ) = \exp \left[ {\alpha_{i} \left( {\theta_{j} - \beta_{ik} } \right)} \right]/\left\{ {1 + \exp \, \left[ {\alpha_{i} \left( {\theta_{j} - \beta_{ik} } \right)} \right]} \right\}$$

where *α*_*i*_ is the discrimination parameter of item *i*, *β*_*ik*_ represents the *k*th difficulty parameter for item *i*, and *θ*_*j*_ is the EPLAD level of person *j*. Each item has an independent discrimination parameter, indicating that the items may differ in their ability to differentiate children with various levels of EPLAD. Different ranges were proposed to better interpret the power of discrimination parameter *α*: 0.01–0.34 = very low; 0.35–0.64 = low; 0.65–1.34 = moderate; 1.35–1.69 = high; and > 1.70 = very high [38]. The difficulty parameter is defined as the level of EPLAD associated with a probability of 50% in response to the category k or higher of an item. GRM allows the spacing between the difficulties of categories to vary across items. The number of difficulty parameters of each item is equal to item categories minus 1. Since ITMAIS is a 5-category Likert scale, 4° of difficulty parameters for each item were produced.

In the present study, both item information and test information, representing the amount of information of each item, and thus the total scale that can provide at a given level of EPLAD was analyzed. In the framework of IRT, item information and test information graphically demonstrates the measurement precision of an item or a scale when assessing subjects with varied levels of EPLAD. The more information could be obtained at a specific level of EPLAD, the higher level of assessment precision and reliability of an item or a scale would be [19]. Therefore, the reliability in the framework of IRT is specified at the item level and combined with individual latent trait.

#### Differential item functioning (DIF) evaluation

DIF analysis aimed to identify discrepancies in responses between children with different genders or different evaluation times, given equivalent levels of EPLAD. In the present study, the iterative hybrid ordinal logistic regression was performed to test DIF of each item. The criterion of an item showing DIF was defined as the magnitude of McFadden pseudo R^2^ > 0.035 [39].

#### Stage 2: Reliability and validity verification of ITMAIS-m

Verification of ITMAIS-m was realized with *SPSS 21.0* and *JASP 0.10.2.0* [40]. Frequencies of missing data, mean score and answer options of each item in ITMAIS-m were calculated. The reliability of ITMAIS-m was analyzed with Cronbach’s α, of which 0.7–0.8 indicates acceptable, 0.8–0.9 indicates good, and above 0.9 represents excellent internal consistency [41]. The item-total correlations of ITMAIS-m were analyzed.

Previous studies have found that hearing grades (classified by PTA) and assessment age would affect the scoring of ITMAIS. Children with more severe hearing loss and younger age would receive lower ITMAIS scores [13, 42]. Therefore, in the aspect of convergent validity analysis, Pearson correlations or Spearman rank-order correlations were applied to explore the relationships of ITMAIS-m with PTA (the better ear) and assessment ages, depending on the distributions of data. The correlations of ITMAIS-m with another childhood auditory outcome measurement (i.e., LEAQ) was also tested. Strength of correlation was evaluated by the correlation coefficient *r*: < 0.3 small, 0.3–0.6 moderate, and > 0.6 large [43].

For known-group validity analysis, the discriminative power of ITMAIS-m among different hearing grades (the better ear) was analyzed by one-way analysis of variance, and effect size among groups was calculated by partial eta squared (η_p_^2^). Furthermore, Bonferroni post hoc tests were performed, and the effect sizes between two groups were quantified by Cohen d. According to the literature, effect size calculated as η_p_^2^ is small when index < 0.01, 0.01–0.06 moderate, and > 0.14 large [44]. The index of d is considered small (0.2–0.5), moderate (0.51–0.8), and large (> 0.8), according to Cohen [45].

## Results

### Characteristics of participants in Stages 1 and 2

Characteristics of the participants recruited in Stages 1 and 2 are summarized in Table 1. The assessment ages in Stage 1 were significantly older, with 1086 individuals assessed with ITMAIS in the follow-up period between 1 month and 4 years after auditory interventions. Children in this stage mostly had the level of profound hearing loss (66.2%) or sensorineural hearing loss (76.3%), while hearing grades in Stage 2 were uniformly distributed. The proportions with conductive (1.3%) and mixed (0.2%) hearing loss in Stage 2 were small, since most cases with the possibility of fluctuated hearing loss were excluded.

**Table 1:** Sample characteristics of Stage 1 and 2

	Stage 1	Stages 2	*p*
Number of participants	1730	450	
Number of ITMAIS/ITMAIS-m assessment	3092	450	
Gender (Male, %)	992(57.3)	268(59.6)	0.422
Assessment age (months, median with IQR) Age ranges (n, %)	29.0(17.6, 41.9)	5.7(3.6, 9.3)	< 0.001
0–12 months	471(15.2)	367(81.6)	
12–24 months	731(23.6)	83(18.4)	
24–36 months	855(27.7)	–	
> 36 months	1035(33.5)	–	
Hearing grades of the better ear (n, %)			< 0.001
Normal	126(7.3)	77(17.1)	
Mild	7(0.4)	49(10.9)	
Moderate	201(11.5)	160(35.6)	
Severe	238(13.8)	54(12.0)	
Profound	1145(66.2)	110(24.4)	
Unknown	13(0.8)	–	
Hearing types of the better ear (n, %)			< 0.001
Sensorineural	1320(76.3)	363(80.7)	
Conductive	226(13.1)	6(1.3)	
Mixed	145(8.3)	1(0.2)	
Unknown	39(2.3)	–	
Auditory interventions (n, %)			
Without any interventions	644(37.2)	450(100)	
Hearing aid	744(43.0)	–	
Cochlear implantation	342(19.8)	–	

*IQR* interquartile range

### Stage 1: Item analysis and modification of ITMAIS

#### Item responses and traditional statistic description

In Stage 1, the percentages of missing answers and response options for each item of ITMAIS are presented in an appendix (see Additional file 2). Percentages of missing answers of the nine items ranged from 0.1 to 2.4%. Inter-item correlations ranged from 0.62 to 0.84.

#### Assumptions checking

EFA demonstrated that the first factor had the largest eigenvalue of 7.01 (accounting for 75% of the variance) with the remainder having eigenvalues less than 1. One factor was thereby extracted, and items loading on the factor ranged from 0.80 to 0.90.

CFA analyzed with a different set of data in Stage 1 revealed a satisfactory 1-factor model fitting except the index of RMSEA (CFI = 0.949, TLI = 0.947, SRMR = 0.030, and RMSEA = 0.134). In comparisons to the 2-factor and 3-factor models, however, they did not ameliorate the model fitting significantly (2-factor model: CFI = 0.971, TLI = 0.969, SRMR = 0.023, and RMSEA = 0.103; 3-factor model: CFI = 0.964, TLI = 0.962, SRMR = 0.026, and RMSEA = 0.118). According to the results obtained from EFA and CFA, the current results indicated that the ITMAIS met the unidimensional assumption.

None of the items violated the assumption of local independence, with residual correlations smaller than 0.10 between items. Likewise, the nine items met the assumption of monotonicity. The relevant graphs plotting item step response function and item response function demonstrated that probabilities of endorsing higher categories in each item increase when auditory function elevates (as shown in the Additional file 3).

#### IRT model fit and parameters evaluation

Five items (item 2, 4, 7, 8, 9) of ITMAIS exhibited unsatisfactory item fit under GRM (*p* < 0.001). In view of the relatively lower factor loading of item 2 (0.80) in unidimensional analysis, it was removed, and re-evaluation demonstrated that only item 9 showed item misfit.

The unidimensionality assumption, item and scale parameters before and after removing item 2 were analyzed and compared. One-factor model fitting of the 8-item ITMAIS (removing item 2), with CFI = 0.946, TLI = 0.924, SRMR = 0.031, RMSEA = 0.154, varied little when compared to the original ITMAIS. Item 2 demonstrated discrimination parameter of 2.380 and item information of 1.758, with difficulty parameters ranging from − 1.583 to 0.590. After removing item 2, the discrimination parameters of the remaining 8 items elevated the largest by 0.232 (item 4), and difficulty parameters fluctuated the most by 0.026 (items 4 and 9). Item information of the remaining 8 items increased from the range of 4.487–8.938 to 4.798–9.259, with the largest elevation of 0.615 in item 4. Test information of the total scale increased from 47.754 to 48.061.

**Fig. 1:**
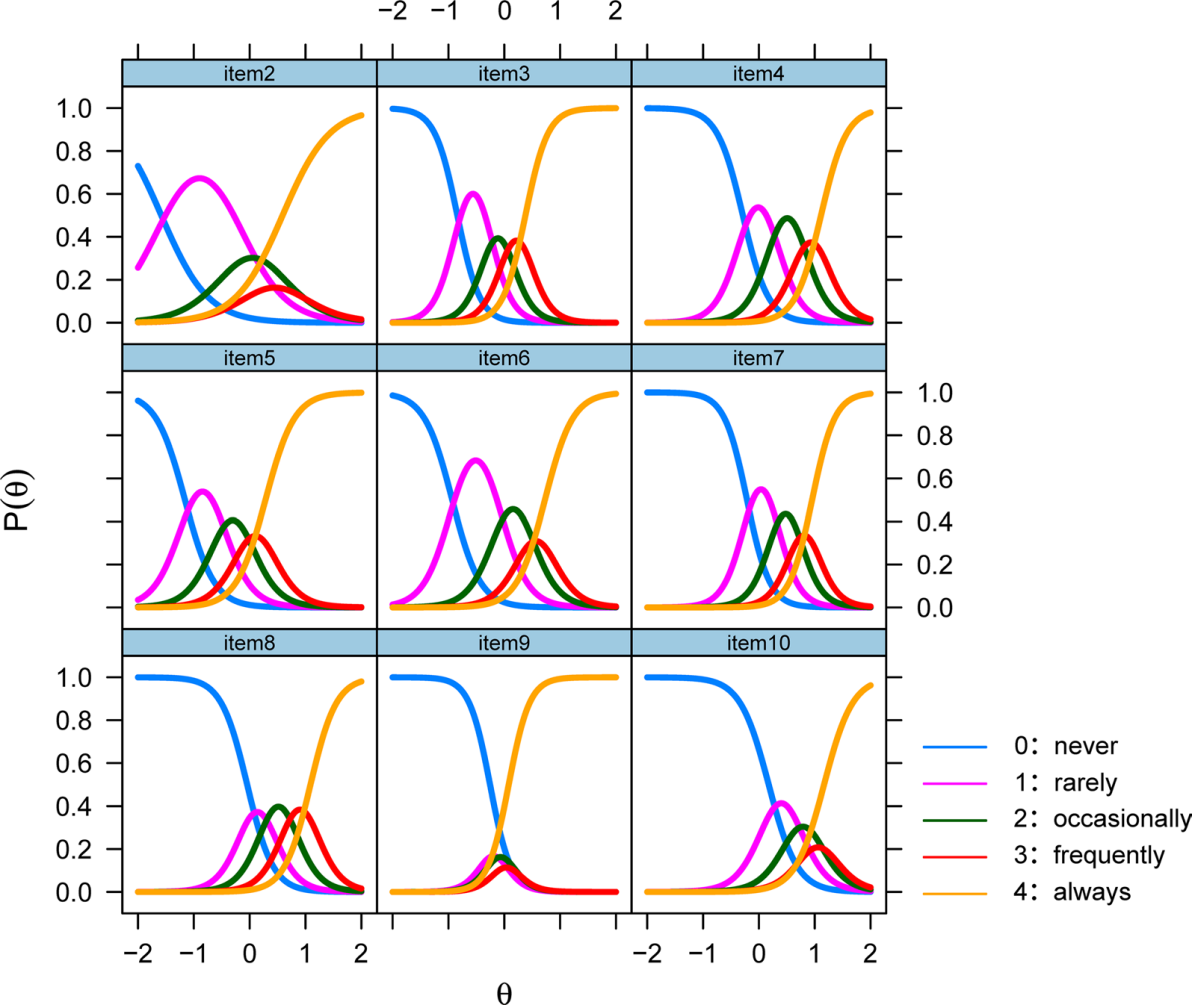
Item trace lines of the 9 items (item 2–10) in ITMAIS. The x axis ‘θ’ represents the range of EPLAD. The y axis ‘P(θ)’ means the probability of an individual with specified EPLAD to respond to different categories of an item

Figure 1 shows the trace lines of each item in ITMAIS. The trace lines demonstrated the probability of selecting a specific response of an item by individuals with a specified level of EPLAD. As shown in the Fig. 1, the response curves of the items were steep and centralized at the EPLAD range of − 1 to 1. It is evident in item 9, showing the response curves were centralized at the EPLAD level of 0. In comparison to other items with orderly response curves, the trace lines of item 2 were relatively poor, showing some of the response curves were disordered and overlapped.

#### Differential item functioning (DIF) evaluation

None of the items in ITMAIS displayed DIF, when individuals presented with different characteristics, i.e., male or female, assessment before or after auditory interventions.

**Fig. 2:**
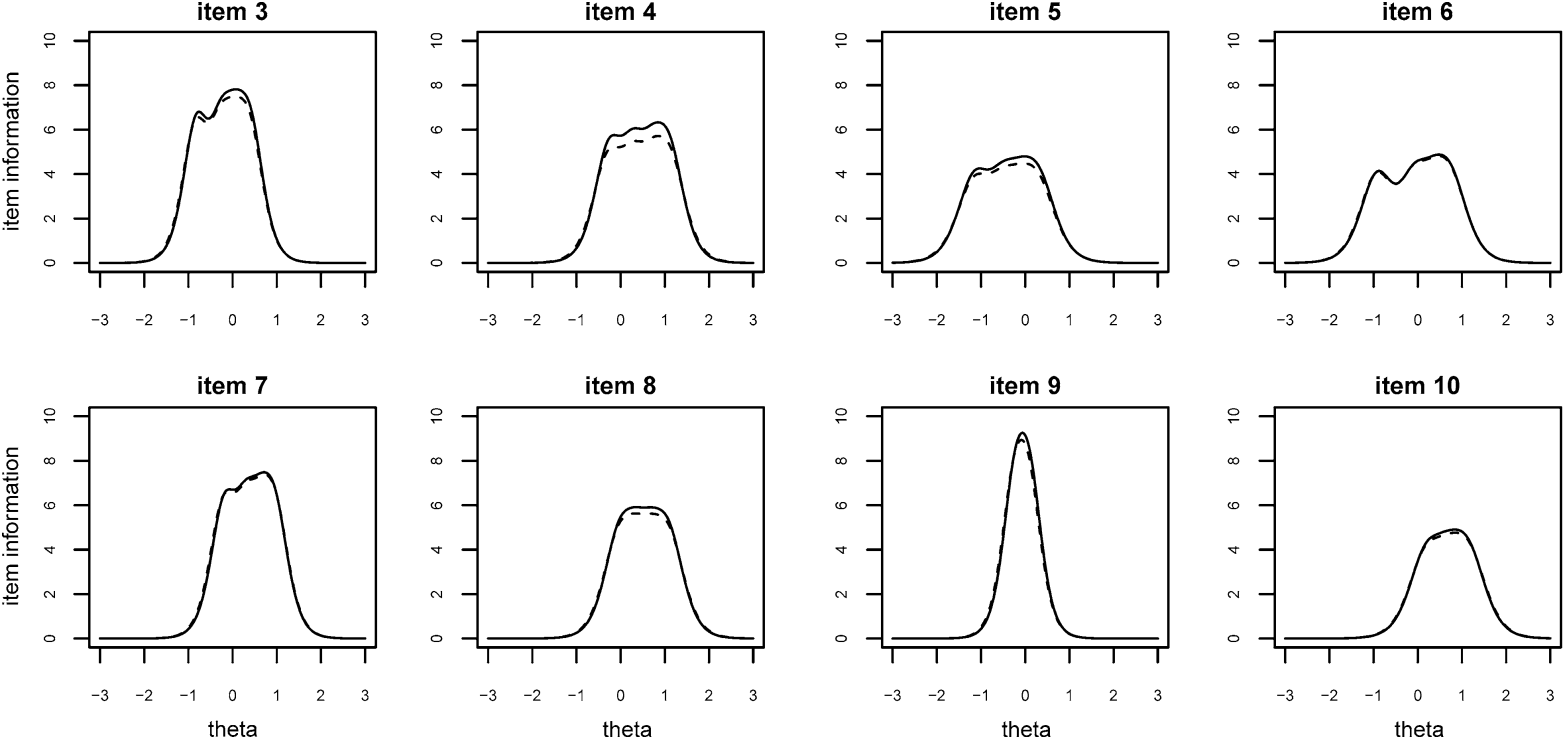
Item information of item 3–10 of ITMAIS, before and after removing item 2. The solid lines represent item information after removing item 2. The dashed lines represent item information without removing item 2. The x axis ‘theta’ represents the range of EPLAD

**Fig. 3:**
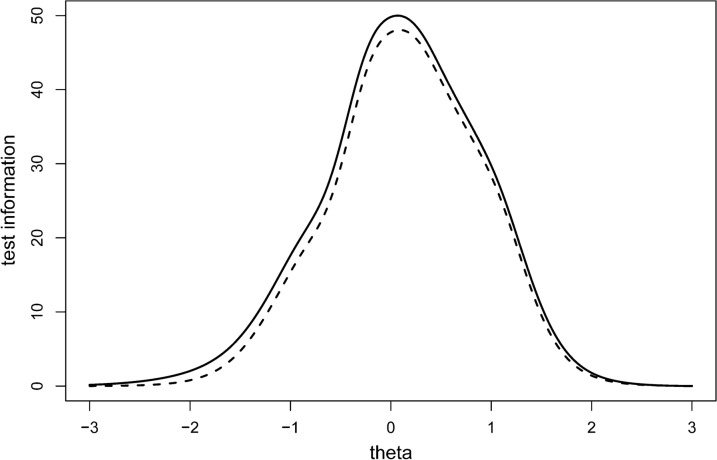
Test information before and after removing item 2. The solid line represents test information of the ITMAIS-m after removing item 2. The dashed line represents test information of ITMAIS without removing item 2. The x axis ‘theta’ represents the range of EPLAD

Ultimately, ITMAIS was modified by removing item 2 in Stage 1. ITMAIS-m demonstrated better item fit, and the item and scale parameters were robust to such modification. Item parameters of ITMAIS-m are presented in Table 2. Item information of item 3–10 and the test information, before and after removing item 2, are plotted in Figs. 2 and 3.

**Table 2:** Estimates of discrimination and difficulty parameters of ITMAIS-m, under the GRM

	Discrimination	Difficulty			
	*α*(SE)	*β1*(SE)	*β2*(SE)	*β3*(SE)	*β4*(SE)
Item 3	5.062(0.169)	− 0.818(0.027)	− 0.256(0.024)	0.073(0.023)	0.390(0.024)
Item 4	4.587(0.148)	− 0.262(0.024)	0.275(0.024)	0.746(0.026)	1.092(0.029)
Item 5	3.957(0.122)	− 1.146(0.031)	− 0.504(0.025)	− 0.059(0.024)	0.295(0.025)
Item 6	3.998(0.121)	− 0.915(0.029)	− 0.073(0.024)	0.414(0.025)	0.728(0.026)
Item 7	4.944(0.163)	− 0.188(0.024)	0.302(0.023)	0.670(0.025)	0.944(0.027)
Item 8	4.394(0.146)	− 0.023(0.024)	0.332(0.023)	0.711(0.026)	1.074(0.030)
Item 9	5.431(0.221)	− 0.229(0.024)	− 0.106(0.023)	0.014(0.023)	0.098(0.023)
Item 10	3.947(0.137)	0.190(0.024)	0.628(0.026)	0.941(0.028)	1.150(0.031)

*ITMAIS-m* modified Infant–Toddler Meaningful Integration Scale, *GRM* graded response model, *SE* standard error

### Stage 2: Reliability and validity verification of ITMAIS-m

Frequencies of missing data, mean score and answer options of each item, as well as item-total correlations in ITMAIS-m were calculated and shown in an appendix (Additional file 4). The item-total correlations of the eight items in ITMAIS-m ranged from 0.693 to 0.851. The ITMAIS-m exhibited excellent internal consistency with Cronbach’s α = 0.919.

As shown in Table 3, correlation of ITMAIS-m with LEAQ was 0.932, suggesting a strong convergence. The correlations with PTA ranged from − 0.670 to − 0.909, and varied in different age groups. ITMAIS-m significantly correlated with assessment ages, when children were with normal hearing, mild, moderate or severe hearing loss, although the power was moderate in children with severe hearing loss (r = 0.380). There was no significant association between ITMAIS-m and assessment ages in children with profound hearing loss.

**Table 3:** Correlations of ITMAIS-m with LEAQ, PTA and age

	n	Mean (SD)/Median (IQR)	ITMAIS-m Mean (SD)/Median (IQR)	r (95% CI)	*p*
Correlations with LEAQ	93	7.9(6.6)	25.5(20.2)	0.932(0.903 to 0.958)	< 0.001
Correlations with PTA (dB HL)					
0–3 months	37	51.5(34.6)	13.9(8.7)	− 0.670(− 0.527 to − 0.790)	< 0.001
3–6 months	207	55.7(35.1)	20.5(13.6)	− 0.719(− 0.666 to − 0.771)	< 0.001
6–9 months	89	53.9(33.6)	32.6(19.2)	− 0.794(− 0.710 to − 0.862)	< 0.001
9–16 months	80	71.4(41.1)	36.9 (31.6)	− 0.909(− 0.866 to − 0.945)	< 0.001
16–24 months	37	73.7(37.5)	31.9(31.6)	− 0.801(− 0.699 to − 0.886)	< 0.001
Correlations with age (m)					
Normal (m)	77	5.1(4.6)	43.0(18.6)	0.776(0.620–0.883)	< 0.001
Mild (m)	49	6.9(4.3)	43.1(23.6)	0.831(0.769–0.902)	< 0.001
Moderate (m)	160	6.8 (4.1)	30.8(19.6)	0.624(0.482–0.763)	< 0.001
Severe (m)	54	7.4(5.4)	14.2(8.1)	0.380(0.084–0.650)	0.005
Profound (m)	110	6.9(9.0)	3.1(4.0)	0.092(− 0.099–0.298)	0.337

*SD* standard deviation, *IQR* interquartile range, *r* correlation coefficient, *CI* confidence interval, *ITMAIS-m* modified Infant–Toddler Meaningful Integration Scale, *LEAQ* LittlEARS Auditory Questionnaire, *PTA* pure-tone average threshold of the better ear

Table 4 demonstrates that children with different hearing grades (normal-mild hearing loss, moderate hearing loss, severe-profound hearing loss) in different age ranges differed significantly in ITMAIS-m scores. The effect sizes η_p_^2^ among groups ranged from 0.515 to 0.844. Post hoc comparisons demonstrated that, excepting comparison between moderate and severe-profound hearing loss within 3 months (Cohen d = 0.41), effect sizes between the other hearing grades in different age ranges were large, with Cohen d ranging from 0.93 to 5.83. The effect sizes of ITMAIS-m were larger when discriminating severe-profound hearing loss from other hearing grades than discriminating between normal hearing-mild hearing loss and moderate hearing loss.

**Table 4:** Known-group validity of ITMAIS-m in discriminating hearing grades in varied age ranges

	n	ITMAIS-m Mean (SD)	F, *p*, η_p_^2^	Post hoc tests^a^ Cohen d (95% CI), *p*		
				Normal-mild versus moderate	Moderate versus severe-profound	Normal-mild versus severe-profound
0–3 months			F(2,34) = 18.03 < .001 0.515	1.57(0.59–2.53) 0.001	0.41(− 0.49–1.29) 0.379	2.12(1.18–3.03) < 0.001
Normal-mild	16	21.0(7.3)				
Moderate	8	9.9 (6.4)				
Severe-profound	13	7.8(4.5)				
3–6 months			F(2,204) = 118.30 < 0.001 0.537	0.96(0.60–1.31) < 0.001	1.76(1.38–2.14) < 0.001	2.98(2.46–3.49) < 0.001
Normal-mild	57	32.7 (10.8)				
Moderate	83	22.7(10.3)				
Severe-profound	67	7.4(5.9)				
6–9 months			F(2,86) = 69.88 < 0.001 0.619	1.28(0.74–1.81) < 0.001	1.83(1.21–2.44) < 0.001	3.70(2.80–4.58) < 0.001
Normal-mild	30	49.6 (11.8)				
Moderate	35	32.9(13.9)				
Severe-profound	24	10.9(8.5)				
9–16 months			F(2,77) = 208.73 < 0.001 0.844	0.93(0.26–1.58) 0.006	4.21(3.29–5.11) < 0.001	5.83(4.61–7.03) < 0.001
Normal-mild	18	73.2(14.7)				
Moderate	22	58.9(16.0)				
Severe-profound	40	8.5 (9.1)				
16–24 months			F(2,34) = 24.19 < 0.001 0.587	1.09(0.01–2.15) 0.048	1.46(0.63–2.28) < 0.001	5.41(3.62–7.16) < 0.001
Normal-mild	6	75.3(8.5)				
Moderate	11	44.6(33.9)				
Severe-profound	20	11.9(12.4)				

*ITMAIS-m* modified Infant–Toddler Meaningful Integration Scale, *SD* standard deviation, *F* analysis of variance, *η*_*p*_^*2*^ partial eta squared, *CI* confidence interval

^a^Bonferroni post hoc tests

## Discussion

The main aim of this research was to modify and verify the ITMAIS—an auditory outcome measurement scale evaluating EPLAD for infants and toddlers—in the framework of psychometric analysis. The research is novel in that it combines modern (IRT) and traditional (CTT) psychometric theories to comprehensively evaluate a scale concentrating on prelingual auditory function. The modified version, ITMAIS-m was found to be reliable and valid tool to evaluate EPLAD in clinical practice precisely and efficiently.

A total of 1730 participants with varied characteristics, including wide age ranges (median (IQR) ages 29.0 (17.6, 41.9) months), different hearing grades and hearing types (normal hearing, or mild to profound hearing grades with sensorineural, conductive or mixed hearing types), and different assessment times (before or after auditory intervention), were recruited in the stage of IRT analysis. The large sample with different characteristics signifies that individuals are with different levels of latent trait, and the widely distributed latent trait covering the whole range enables accurate and stable item and scale parameters estimation with lower standard error [20, 46]. Barker et al. [47] has tried to use Rasch; i.e., a one-parameter IRT model, to examine the psychometric properties of ITMAIS. Their conclusions, however, may deserve further discussion as a result of the limitation imposed by the small, homogenous and tailored sample of 23 cochlear implanted children with severe to profound sensorineural hearing loss.

In the present study, GRM model fitting demonstrated that five items were poorly fitted. In view of item content, item 2 (*Does the child produce well-formed syllables and syllable-sequences that are recognized as speech?*) mainly evaluates preverbal vocalization, which differs from the nature of EPLAD. In addition, the results of the poor performance of trace lines of item 2, the minor variations of dimensionality and item parameters after deletion, as well as ameliorated GRM model fitting after deletion, indicates it is appropriate to modify ITMAIS by removing item 2.

Although the GRM model fitting of the 7-item ITMAIS, removing both item 2 and 9, is preferable, the plunge of test information (from 48.061 to 40.216) and the highest information provided by item 9 (9.259) suggests it is not advisable to modify ITMAIS by removing item 9, with the possible loss of a large amount of information. Moreover, the content of item 9 (*Does the child spontaneously know the difference between speech and non-speech stimuli with listening alone?*) largely reflects the function of sound discrimination and identification, which is highly related to the nature of EPLAD. Given that no optimal fit indices exist, it is recommended that strict IRT model fitting is not vital, and some unsatisfactorily fitted items may be retained if identified with a close clinical relationship [29].

To date, there are few studies that concentrate on IRT analysis of scales evaluating EPLAD, although the EPLAD is fundamental and vital to speech and language development [47, 48]. IRT is an accessible way to develop or modify a scale focusing on item responses. Good performed items, with adequate model fit, high discriminative power, appropriate difficulty range and no signs of DIF, could be selected out through this approach. In the present study, we have identified that ITMAIS-m assesses the sole latent trait, i.e., EPLAD, with the method of unidimensionality checking. Each item had a very high discriminative power (α > 1.70), and the 8 items of ITMAIS-m demonstrated difficulty span covering the level of EPLAD from − 1.146 to + 1.150, implying that ITMAIS-m is robust in discriminating an individual with EPLAD below or above the mean level (θ = 0). Considering the difficulty range of the items in ITMAIS-m is not wide enough to cover the full range of EPLAD, it would be a further direction to research on widening the difficulty range of ITMAIS by adding more items.

As shown in Fig. 3, the maximum test information of ITMAIS-m reaches to 48.061. With the formula that reliability = 1–1/test information, the reliability of ITMAIS-m could reach the highest level of 0.979 when evaluating children with EPLAD approaches approximately the mean level (θ = 0) [49]. This is consistent with the results obtained from the analysis in Stage 2, in which the Cronbach’s α of ITMAIS-m was 0.919. Considering the centralized tendency of the test information, the results indicate that ITMAIS-m would provide sufficient information when assessing children with EPLAD approximately distributed between − 1.3 and 1.5 SD. Within this range, the ITMAIS-m could provide test information more than 10, and the reliability of ITMAIS-m could reach 0.90 or higher accordingly by conversion.

In Stage 2, analysis based on CTT was used to verify the psychometric properties of ITMAIS-m with a separate specific sample. By exerting the superiority of CTT in evaluating external construct validity of a scale, the relationship of ITMAIS-m with LEAQ, age, as well as clinical characteristics were evaluated. Apart from the high correlations with LEAQ, ITMAIS-m was significantly correlated with PTA. The older the children, the higher the correlations between ITMAIS-m and PTA. This phenomenon can be seen from previous studies where the increase of ITMAIS scoring slows down when children grow older, implying that age also affects ITMAIS scoring and EPLAD [42]. However, when children grow older, the effect of age on ITMAIS is minor, and the relationship between ITMAIS-m and PTA becomes more robust. This is also the reason why ITMAIS-m simultaneously correlates with age in children with different hearing grades, except those with profound hearing loss. In the approach of known-group validity evaluation, ITMAIS-m could efficiently discriminate different hearing grades in different age groups, especially distinguishing severe-profound hearing loss from other hearing grades. Considering the high correlation with PTA and significant discriminative power in hearing grades, the value of ITMAIS-m in predicting hearing grades, especially in children with severe and profound hearing loss who are crying for auditory diagnosis and intervention, could be further investigated.

There are a few limitations in the present study. The number of participants in Stage 2 within 3 months and 16 months or larger is relatively limited, which results in an instability of parameter evaluation in the subgroup of 0–3 months and 16–24 months. In view of the main purpose of analyzing construct validity by evaluating the relationships between ITMAIS-m and hearing grades, the sample included in Stage 2 only concerns individuals without auditory intervention. In future, larger samples with different clinical characteristics, e.g., different forms and periods of auditory intervention, could be included to further verify the validity of ITMAIS-m.

## Conclusions

With the comprehensive and complementary approach of combining IRT and CTT, the modified ITMAIS is developed to have robust psychometric properties. This important result indicates the significance and benefit of using IRT in combination with CTT in modifying auditory outcome measurement scales. Moreover, the ITMAIS-m obtained from the present study will provide a useful clinical tool to evaluate EPLAD for young children more precisely and efficiently. Further research is currently underway to validate the clinical applications of ITMAIS-m in predicting young children’s hearing grades when audiometry was unavailable.

## Supplementary information

**Additional file 1.** The content of ITMAIS (without item 1).

**Additional file 2. **Responses on the 9-item ITMAIS at Stage 1.

**Additional file 3.** The item step response function and item response function of items in ITMAIS.

**Additional file 4.** Item responses and item-total correlations of ITMAIS-m at Stage 2.

## Data Availability

Data is available on request from corresponding author.

## Abbreviations

EPLAD: Early prelingual auditory development
ITMAIS: Infant–Toddler Meaningful Auditory Integration Scale
ITMAIS-m: Modified Infant–Toddler Meaningful Auditory Integration Scale
IRT: Item response theory
CTT: Classical test theory
JCIH: Joint Committee on Infant Hearing
EHDI: Early Hearing Detection and Intervention
PTA: Pure tone average threshold
IQR: Interquartile range
LEAQ: LittlEARS Auditory Questionnaire
EFA: Exploratory factor analysis
CFA: Confirmatory factor analysis
CFI: Comparative fit index
TLI: Tucker–Lewis index
RMSEA: Root mean square error of approximation
SRMR: Standardized root mean residuals
GRM: Graded response model
DIF: Differential item functioning

## References

[1] World Health Organization. Deafness and hearing loss; 2020. https://www.who.int/news-room/fact-sheets/detail/deafness-and-hearing-loss. Accessed 8 Nov 2020.

[2] Moeller MP (2000). Early intervention and language development in children who are deaf and hard of hearing. Pediatrics.

[3] Stika CJ, Eisenberg LS, Johnson KC, Henning SC, Colson BG, Ganguly DH (2015). Developmental outcomes of early-identified children who are hard of hearing at 12 to 18 months of age. Early Hum Dev.

[4] Reed NS, Altan A, Deal JA, Yeh C, Kravetz AD, Wallhagen M (2019). Trends in health care costs and utilization associated with untreated hearing loss over 10 years. JAMA Otolaryngol Head Neck Surg.

[5] Joint Committee on Infant Hearing, American Academy of Audiology, American Academy of Pediatrics, American Speech-Language-Hearing Association, Directors of Speech and Hearing Programs in State Health and Welfare Agencies (2000). Year 2000 position statement: principles and guidelines for early hearing detection and intervention programs. Pediatrics.

[6] Joint Committee on Infant Hearing (2007). Year 2007 position statement: principles and guidelines for early hearing detection and intervention programs. Pediatrics.

[7] Joint Committee on Infant Hearing (2019). Year 2019 position statement: principles and guidelines for early hearing detection and intervention programs. J Early Hear Detect Interv.

[8] Bagatto MP, Moodie ST, Seewald RC, Bartlett DJ, Scollie SD (2011). A critical review of audiological outcome measures for infants and children. Trends Amplif.

[9] Welling D, Ukstins CA, Welling Deborach R, Ukstins Carol A (2017). Fundamentals of audiology for the speech-language pathologist. Understanding auditory development and the child with hearing loss.

[10] Eisenberg LS, Johnson KC, Martinez AS, Cokely CG, Tobey EA, Quittner AL (2006). Speech recognition at 1-year follow-up in the childhood development after cochlear implantation study: methods and preliminary findings. Audiol Neurootol.

[11] Ben-Itzhak D, Greenstein T, Kishon-Rabin L (2014). Parent report of the development of auditory skills in infants and toddlers who use hearing aids. Ear Hear.

[12] Pelosi S, Wanna G, Hayes C, Sunderhaus L, Haynes DS, Bennett ML (2013). Cochlear implantation versus hearing amplification in patients with auditory neuropathy spectrum disorder. Otolaryngol Head Neck Surg.

[13] Zheng Y, Soli SD, Wang K, Meng J, Meng Z, Xu K (2009). A Normative study of early prelingual auditory development. Audiol Neurootol.

[14] Kishon-Rabin L, Taitelbaum R, Elichai O, Maimon D, Debyiat D, Chazan N (2001). Developmental aspects of the IT-MAIS in normal-hearing babies. Isr J Speech Hear.

[15] Weichbold V, Anderson I, D'Haese P (2004). Validation of three adaptations of the Meaningful Auditory Integration Scale (MAIS) to German. Engl Pol Int J Audiol.

[16] Cavicchiolo S, Mozzanica F, Guerzoni L, Murri A, Dall'Ora I, Ambrogi F (2018). Early prelingual auditory development in Italian infants and toddlers analysed through the Italian version of the Infant-Toddler Meaningful Auditory Integration Scale (IT-MAIS). Eur Arch Oto-Rhino-L.

[17] Zhong Y, Xu T, Dong R, Lyu J, Liu B, Chen X (2017). The analysis of reliability and validity of the IT-MAIS, MAIS and MUSS. Int J Pediatr Otorhinolaryngol.

[18] Thomas ML (2011). The value of item response theory in clinical assessment: a review. Assessment.

[19] De Champlain AF (2010). A primer on classical test theory and item response theory for assessments in medical education. Med Educ.

[20] Cappelleri JC, Jason Lundy J, Hays RD (2014). Overview of classical test theory and item response theory for the quantitative assessment of items in developing patient-reported outcomes measures. Clin Ther.

[21] Kimberlin CL, Winterstein AG (2008). Validity and reliability of measurement instruments used in research. Am J Health Syst Pharm.

[22] Heffernan E, Maidment DW, Barry JG, Ferguson MA (2019). Refinement and validation of the Social Participation Restrictions Questionnaire: an application of Rasch analysis and traditional psychometric analysis techniques. Ear Hear.

[23] Reise SP, Waller NG (2009). Item response theory and clinical measurement. Annu Rev Clin Psychol.

[24] Reise SP, Haviland MG (2005). Item response theory and the measurement of clinical change. J Pers Assess.

[25] Chachamovich E, Fleck MP, Trentini CM, Laidlaw K, Power MJ (2008). Development and validation of the Brazilian version of the Attitudes to Aging Questionnaire (AAQ): an example of merging classical psychometric theory and the Rasch measurement model. Health Qual Life Outcomes.

[26] Hughes SE, Rapport F, Watkins A, Boisvert I, McMahon CM, Hutchings HA (2019). Study protocol for the validation of a new patient-reported outcome measure (PROM) of listening effort in cochlear implantation: the Listening Effort Questionnaire-Cochlear Implant (LEQ-CI). BMJ Open.

[27] Coninx F, Weichbold V, Tsiakpini L, Autrique E, Bescond G, Tamas L (2009). Validation of the LittlEARS((R)) Auditory Questionnaire in children with normal hearing. Int J Pediatr Otorhi.

[28] World Health Organization. Grades of hearing impairment; 2015. https://www.who.int/pbd/deafness/hearing_impairment_grades/en/. Accessed 8 Nov 2020.

[29] Reeve BB, Hays RD, Bjorner JB, Cook KF, Crane PK, Teresi JA (2007). Psychometric evaluation and calibration of health-related quality of life item banks. Med Care.

[30] R Core Team. The R Project for Statistical Computing; 2018. https://www.r-project.org/. Accessed 8 Nov 2020.

[31] Kawata AK, Hareendran A, Shaffer S, Mannix S, Thach A, Desai P (2019). Evaluating the Psychometric Properties of the Migraine Functional Impact Questionnaire (MFIQ). Headache.

[32] Hu L, Bentler PM (1999). Cutoff criteria for fit indexes in covariance structure analysis: conventional criteria versus new alternatives. Struct Equ Model Multidiscip J.

[33] Khazaal Y, Breivik K, Billieux J, Zullino D, Thorens G, Achab S (2018). Game Addiction Scale Assessment through a nationally representative sample of young adult men: item response theory graded-response modeling. J Med Internet Res.

[34] Mokkink LB, Galindo-Garre F, Uitdehaag BM (2016). Evaluation of the Multiple Sclerosis Walking Scale-12 (MSWS-12) in a Dutch sample: application of item response theory. Mult Scler.

[35] Embretson SE, Reise SP (2000). Item response theory for psychologists.

[36] Samejima F (1969). Estimation of latent ability using a response pattern of graded scores. Psychometrika.

[37] Chiarotto A, Bishop A, Foster NE, Duncan K, Afolabi E, Ostelo RW (2018). Item response theory evaluation of the biomedical scale of the Pain Attitudes and Beliefs Scale. PLoS ONE.

[38] Baker FB, Kim SH (2017). The basics of item response theory using R.

[39] Jodoin MG, Gierl MJ (2001). Evaluating type I error and power rates using an effect size measure with the logistic regression procedure for DIF detection. Appl Meas Educ.

[40] JASP Team. JASP: a fresh way to do statistics; 2018. https://jasp-stats.org/. Accessed 8 Nov 2020.

[41] Cronbach LJ (1951). Coefficient alpha and the internal structure of tests. Psychometrika.

[42] Liang S, Soli SD, Zheng Y, Li G, Meng Z (2016). Initial classification of pediatric hearing impairment using behavioral measures of early prelingual auditory development. Int J Audiol.

[43] Hinkle D, Wiersma W, Jurs S (2003). Applied Statistics for the behavioral sciences.

[44] Blasco-Bonora PM, Martín-Pintado-Zugasti A (2017). Effects of myofascial trigger point dry needling in patients with sleep bruxism and temporomandibular disorders: a prospective case series. Acupunct Med.

[45] Cohen J (1988). Statistical power analysis for the behavioral sciences.

[46] Walker J, Bohnke JR, Cerny T, Strasser F (2010). Development of symptom assessments utilising item response theory and computer-adaptive testing—a practical method based on a systematic review. Crit Rev Oncol Hematol.

[47] Barker BA, Donovan NJ, Schubert AD, Walker EA (2017). Using Rasch analysis to examine the item-level psychometrics of the Infant–Toddler Meaningful Auditory Integration Scales. Speech Lang Hear.

[48] Keilmann A, Friese B, Hoffmann V (2019). Receptive and productive speech and language abilities in hearing-impaired children with German as a second language. Int J Pediatr Otorhi.

[49] Reeve BB, Madans J, Miller K, Maitland A, Willis G (2011). Applying Item Response Theory for Questionnaire evaluation. Question evaluation methods: contributing to the science of data quality.

