# Supplementary material for: Modification and verification of the Infant–Toddler Meaningful Auditory Integration Scale: a psychometric analysis combining item response theory with classical test theory
Source: Health Qual Life Outcomes. 2020 Nov 13;18:367. doi: 10.1186/s12955-020-01620-9 (PMC7663878; doi:10.1186/s12955-020-01620-9)
Supplement: Supplementary file 1 — Additional file 1. The content of ITMAIS (without item 1). [file 12955_2020_1620_MOESM1_ESM.docx]

The content of ITMAIS (without item 1)*.

|  | **Content** | **score** |
| --- | --- | --- |
| **Item 2** | Does the child produce well-formed syllables and syllable-sequences that are recognized as speech? | 0 1 2 3 4 |
| **Item 3** | Does the child spontaneously respond to his/her name in quiet with auditory cues only when not expecting to hear it? | 0 1 2 3 4 |
| **Item 4** | Does the child spontaneously respond to his/her name in the presence of background noise with auditory cues only? | 0 1 2 3 4 |
| **Item 5** | Does the child spontaneously alert to environmental sounds in the home without being told or prompted to do so? | 0 1 2 3 4 |
| **Item 6** | Does the child spontaneously alert to environmental sounds in new environments? | 0 1 2 3 4 |
| **Item 7** | Does the child spontaneously recognize auditory signals that are part of his/her everyday routines? | 0 1 2 3 4 |
| **Item 8** | Does the child demonstrate the ability to discriminate spontaneously between two speakers with auditory cues only? | 0 1 2 3 4 |
| **Item 9** | Does the child spontaneously know the difference between speech and non-speech stimuli with listening alone? | 0 1 2 3 4 |
| **Item 10** | Does the child spontaneously associate vocal tone with its meaning based on hearing alone? | 0 1 2 3 4 |

*referring to Zimmerman-Phillips S OM, Robbins AM. Infant-Toddler: Meaningful Auditory Integration Scale(IT-MAIS). Sylmer, Calif: Advanced Bionics Corporation. 1997.
