# Supplementary material for: Modification and verification of the Infant–Toddler Meaningful Auditory Integration Scale: a psychometric analysis combining item response theory with classical test theory
Source: Health Qual Life Outcomes. 2020 Nov 13;18:367. doi: 10.1186/s12955-020-01620-9 (PMC7663878; doi:10.1186/s12955-020-01620-9)
Supplement: Supplementary file 2 — Additional file 2. Responses on the 9-item ITMAIS at Stage 1. [file 12955_2020_1620_MOESM2_ESM.docx]

**Additional file 2 Responses on the 9-item ITMAIS at Stage 1.**

| **items** | **Non-missing (n, %)** | **Mean (SD) Scores** | **Answer options (n, %)** | | | | |
| --- | --- | --- | --- | --- | --- | --- | --- |
|  |  |  | **0** | **1** | **2** | **3** | **4** |
| **Item 2** | 3397(99.8%) | 2.16(1.44) | 355(10.5) | 1165(34.3) | 527(15.5) | 274(8.1) | 1076(31.7) |
| **Item 3** | 3402(99.9%) | 2.24(1.61) | 748(22.0) | 602(17.7) | 385(11.3) | 410(12.1) | 1257(36.9) |
| **Item 4** | 3363(98.8%) | 1.42(1.46) | 1339(39.8) | 626(18.6) | 554(16.5) | 349(10.4) | 495(14.7) |
| **Item 5** | 3398(99.8%) | 2.45(1.50) | 482(14.2) | 606(17.8) | 541(15.9) | 435(12.8) | 1334(39.3) |
| **Item 6** | 3358(98.6%) | 1.94(1.48) | 677(20.2) | 910(27.1) | 569(16.9) | 345(10.3) | 857(25.5) |
| **Item 7** | 3351(98.4%) | 1.44(1.55) | 1432(42.7) | 574(17.1) | 430(12.8) | 284(8.5) | 631(18.8) |
| **Item 8** | 3323(97.6%) | 1.35(1.55) | 1601(48.2) | 392(11.8) | 424(12.8) | 362(10.9) | 544(16.4) |
| **Item 9** | 3338(98.0%) | 2.19(1.89) | 1320(39.5) | 126(3.8) | 135(4.0) | 110(3.3) | 1647(49.3) |
| **Item 10** | 3371(99.0%) | 1.08(1.47) | 1897(56.3) | 480(14.2) | 313(9.3) | 190(5.6) | 491(14.6) |

SD: standard deviation
