# Supplementary material for: Modification and verification of the Infant–Toddler Meaningful Auditory Integration Scale: a psychometric analysis combining item response theory with classical test theory
Source: Health Qual Life Outcomes. 2020 Nov 13;18:367. doi: 10.1186/s12955-020-01620-9 (PMC7663878; doi:10.1186/s12955-020-01620-9)
Supplement: Supplementary file 4 — Additional file 4. Item responses and item-total correlations of ITMAIS-m at Stage 2. [file 12955_2020_1620_MOESM4_ESM.docx]

**Item responses and item-total correlations of ITMAIS-m at Stage 2.**

| **item** | **Non-missing (n, %)** | **Mean (SD) Scores** | **Answer options (n, %)** | | | | | **Item-total correlation** |
| --- | --- | --- | --- | --- | --- | --- | --- | --- |
|  |  |  | **0** | **1** | **2** | **3** | **4** |  |
| **Item 3** | 448(99.6%) | 1.50(1.25) | 109(24.3) | 151(33.7) | 86(19.2) | 61(13.6) | 41(9.2) | 0.848 |
| **Item 4** | 407(90.4%) | 0.84(1.00) | 195(47.9) | 122(30.0) | 59(14.5) | 24(5.9) | 7(1.7) | 0.851 |
| **Item 5** | 449(99.8%) | 2.34(1.34) | 39(8.7) | 105(23.4) | 96(21.4) | 81(18.0) | 128(28.5) | 0.800 |
| **Item 6** | 438(97.3%) | 1.38(1.21) | 92(21.0) | 191(43.6) | 91(20.8) | 33(7.5) | 29(6.6) | 0.803 |
| **Item 7** | 448(99.6%) | 0.66(0.93) | 256(57.1) | 121(27.0) | 46(10.3) | 17(3.8) | 8(1.9) | 0.817 |
| **Item 8** | 443(98.4%) | 0.62(0.95) | 270(60.9) | 107(24.2) | 37(8.4) | 21(4.7) | 8(1.8) | 0.835 |
| **Item 9** | 445(98.9%) | 0.73(1.24) | 291(65.4) | 72(16.2) | 30(6.7) | 15(3.4) | 37(8.3) | 0.829 |
| **Item 10** | 440(97.8%) | 0.25(0.57) | 356(80.9) | 65(14.8) | 15(3.4) | 3(0.7) | 1(0.2) | 0.693 |

SD: standard deviation
