# Supplementary material for: Modification and verification of the Infant–Toddler Meaningful Auditory Integration Scale: a psychometric analysis combining item response theory with classical test theory
Source: Health Qual Life Outcomes. 2020 Nov 13;18:367. doi: 10.1186/s12955-020-01620-9 (PMC7663878; doi:10.1186/s12955-020-01620-9)
Supplement: Supplementary file 3 — Additional file 3. The item step response function and item response function of items in ITMAIS. [file 12955_2020_1620_MOESM3_ESM.pdf]

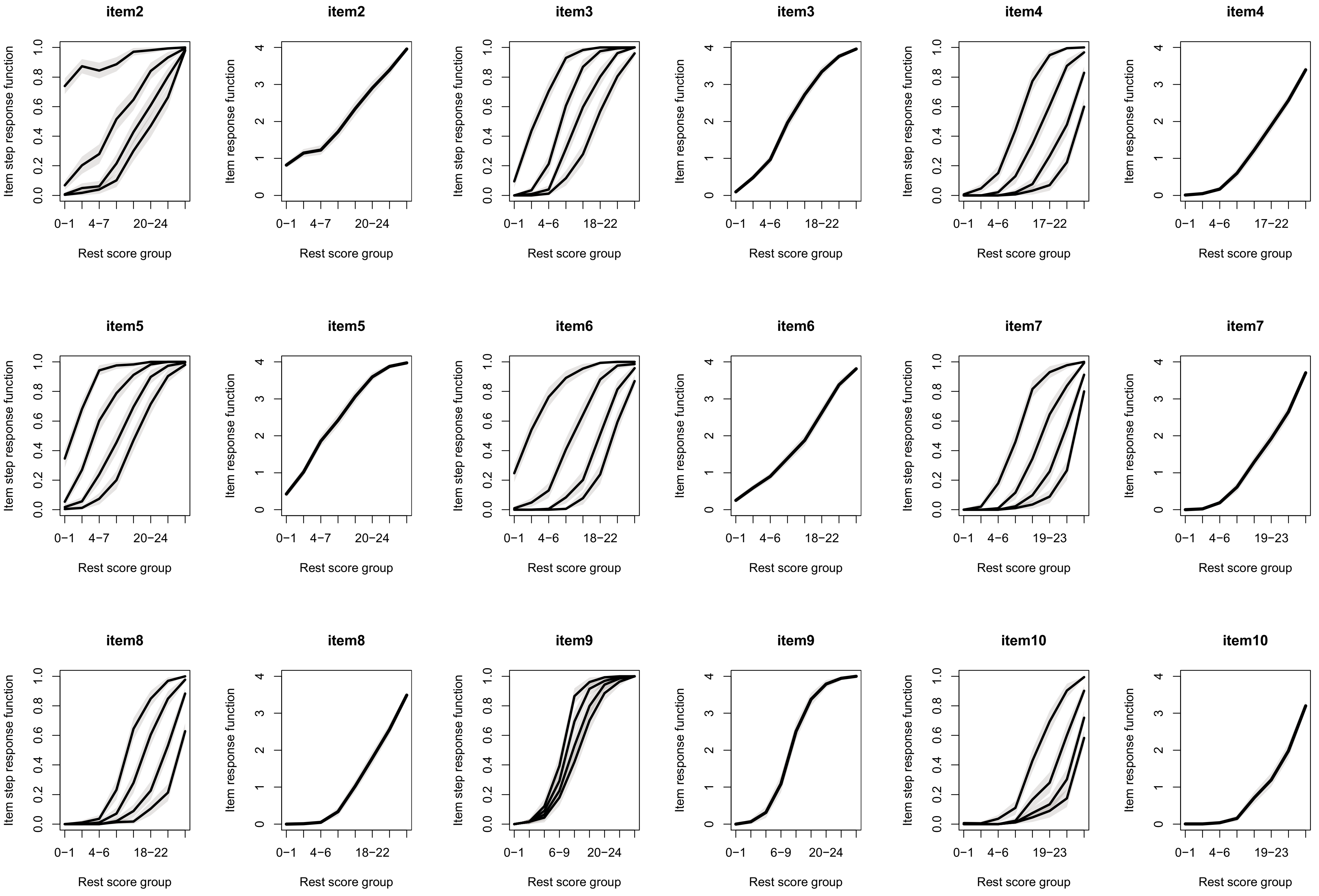
item2
Item step response function
0.0 0.2 0.4 0.6 0.8 1.0
0–1 4–7 20–24
Rest score group
item2
Item response function
0 1 2 3 4
0–1 4–7 20–24
Rest score group
item3
Item step response function
0.0 0.2 0.4 0.6 0.8 1.0
0–1 4–6 18–22
Rest score group
item3
Item response function
0 1 2 3 4
0–1 4–6 18–22
Rest score group
item4
Item step response function
0.0 0.2 0.4 0.6 0.8 1.0
0–1 4–6 17–22
Rest score group
item4
Item response function
0 1 2 3 4
0–1 4–6 17–22
Rest score group
item5
Item step response function
0.0 0.2 0.4 0.6 0.8 1.0
0–1 4–7 20–24
Rest score group
item5
Item response function
0 1 2 3 4
0–1 4–7 20–24
Rest score group
item6
Item step response function
0.0 0.2 0.4 0.6 0.8 1.0
0–1 4–6 18–22
Rest score group
item6
Item response function
0 1 2 3 4
0–1 4–6 18–22
Rest score group
item7
Item step response function
0.0 0.2 0.4 0.6 0.8 1.0
0–1 4–6 19–23
Rest score group
item7
Item response function
0 1 2 3 4
0–1 4–6 19–23
Rest score group
item8
Item step response function
0.0 0.2 0.4 0.6 0.8 1.0
0–1 4–6 18–22
Rest score group
item8
Item response function
0 1 2 3 4
0–1 4–6 18–22
Rest score group
item9
Item step response function
0.0 0.2 0.4 0.6 0.8 1.0
0–1 6–9 20–24
Rest score group
item9
Item response function
0 1 2 3 4
0–1 6–9 20–24
Rest score group
item10
Item step response function
0.0 0.2 0.4 0.6 0.8 1.0
0–1 4–6 19–23
Rest score group
item10
Item response function
0 1 2 3 4
0–1 4–6 19–23
Rest score group
